# Chemical templates

**DOI:** 10.3762/bjoc.10.174

**Published:** 2014-07-22

**Authors:** Sigurd Höger

**Affiliations:** 1 Kekulé-Institut für Organische Chemie und Biochemie, Rheinische Friedrich-Wilhelms-Universität Bonn, Gerhard-Domagk-Str. 1, 53121 Bonn, Germany

**Keywords:** template

When a single object is created from its constituents, a step-by-step assembly strategy is a modus operandi which allows the control of success and sometimes even an error correction after each sequential step. Moreover, it guarantees the uniqueness of the fabricated object. However, the fabrication of several identical objects by assembly or by shaping its constituents is much more effective if a mold or template can be used. All this holds true for the macroscopic as well as for the microscopic world.

A chemical template controls a reaction in such a way that from a multitude of possible products a specific one is preferentially or even exclusively formed. The template can influence the reaction in a stoichiometric or a catalytic way, it can be reused or remain in the product as an integral part.

Template effects in chemistry are commonly associated with the formation of (macro)cycles, or covalent bond formation in general. However, chemical templates can also exert a variety of other effects. They can influence the formation of non-covalent bonds or they can control the positioning of objects in one, two or three dimensions. For example, a functionalized linear template strand leads to the generation of a complementary functionalized product strand. A two-dimensional template, often a planar substrate with a specific structure and composition, determines the respective pattern formation on its surface. And three-dimensional templates are a unique and versatile tool for providing well-designed volume structures, often decorated with useful functionality. Not surprisingly, the annual publication activity of scientific articles in the chemical community containing the concept “template” has doubled within the last decade and is expected to increase further.

Remarkable breakthroughs in synthetic, spectroscopic and microscopic as well as theoretical methods facilitate a closer investigation and more detailed understanding of template effects in all areas of chemistry. It will also lead to the identification of new template effects and new methodologies for the adaptation of existing binding motifs into functional chemical matrices. The articles in this Thematic Series represent a snapshot of the current activities in template-directed reactions. This selection from a broad variety of different fields of chemistry will hopefully be inspiring for the reader, thus leading to new exciting discoveries in template chemistry and attract new audience to this field.

Sigurd Höger

Bonn, June 2014

